# Editorial: Linking Ecosystem Function to Microbial Diversity

**DOI:** 10.3389/fmicb.2016.01041

**Published:** 2016-06-30

**Authors:** Anne E. Bernhard, John J. Kelly

**Affiliations:** ^1^Biology Department, Connecticut CollegeNew London, CT, USA; ^2^Biology Department, Loyola University ChicagoChicago, IL, USA

**Keywords:** nitrogen, nitrification, DNRA, microbialites, metagenomics, methane seeps, stable isotope probing, metacommunity

Understanding the link between microbial diversity and ecosystem processes is a fundamental goal of microbial ecologists, yet we still have a rudimentary knowledge of how changes in diversity affect nutrient cycling and energy transfer in ecosystems. Due to the complexity of the problem, many published studies on this topic have been conducted in artificial or manipulated systems. Studies of artificial assemblages of microbial species have shown direct links between species evenness and ecosystem stability (Wittebolle et al., 2009), and the importance of complementarity for resource use among taxa (Salles et al.). Others have taken a microcosm approach. Webster et al. (2005) added nitrogen to soil microcosms and showed a link between ammonia-oxidizing bacterial diversity and nitrification rates, while Philippot et al. (2013) used a dilution approach to elucidate impacts of the loss of diversity of denitrifying communities on denitrification rates. One commonality of these studies is the focus on specific functional traits and functional gene sequences, which may serve as better proxies of the relationship between diversity and function, compared to more general phylogenetic markers of diversity. In this Research Topic, researchers expand on this theme by conducting transplantation experiments to study metacommunity dynamics in arctic lakes (Adams et al.), using mesocosms to quantify the relationship between nitrifiers and nitrification rates (Bowen et al.), and challenging long-held paradigms about the impacts of nutrient supply and phytoplankton community composition (Glibert et al.).

Although these approaches have allowed researchers to begin to elucidate some possible mechanisms governing ecosystem function and diversity, it is still a challenge to produce conclusive results that relate directly to natural systems. The few studies that have explored the link between diversity and activity in natural systems have typically focused on specific nutrient cycles or processes, such as nitrification, denitrification, and organic carbon degradation pathways, and the microbes that mediate them. For example, Cavigelli and Robertson (2000) found that soil denitrifying communities from tilled agricultural soils respond differently to environmental changes compared to native soils, and Kelly et al. (2011) reported that communities of ammonia-oxidizing archaea and ammonia-oxidizing bacteria respond differently to soil amendments, with ammonia-oxidizing archaea most strongly linked to nitrification rates. These studies emphasize the importance of native microbial communities in responding to environmental changes and modulating critical biochemical processes. Similarly, Bernhard et al. (2007) found functionally distinct ammonia-oxidizing bacterial communities along an estuarine salinity gradient, providing another direct, and quantitative link between microbial biodiversity and ecosystem function. Not surprisingly, many of the studies in this Research Topic focus again on nitrogen-cycling processes in natural systems and how the diversity of functional genes relates to the dominance of specific processes under different environmental conditions. Song et al. provide one of the first studies to show that diversity of bacteria that carry out dissimilatory nitrate reduction to ammonium (DNRA) may have significant impacts on DNRA rates, ultimately controlling the fate of nitrogen in estuaries. Damashek et al. report data from the nutrient-rich regions of San Francisco Bay suggesting that community structure of nitrifiers and biogeochemical function respond to differences in nutrient loading. And in a departure from the N cycle, Marlow et al. help to elucidate drivers of community structure and function at deep-sea methane seeps, and provide insight into differences in elemental cycling between archaeal and bacterial communities.

Earlier studies of microbial diversity and processes in natural systems provide a framework for additional studies to broaden our understanding of the role of microbial diversity in ecosystem function. The problem is complex, but with recent advances in sequencing technology, -omics, and *in-situ* measurements of ecosystem processes, and their applications to microbial communities, making direct connections between ecosystem function and microbial diversity seems more tractable than ever. In this Research Topic, metagenomic analysis of microbialites reveals novel functional guilds distinct from surrounding water and sediment (White et al.). In a companion study of an anthropogenic microbialite-forming ecosystem, White et al. again use metagenomic approaches to identify metabolic drivers of microbialite formation in the subarctic. Using stable isotope probing (SIP), Darjany et al. identify specific bacteria capable of using lignocellulose from salt marsh plants, representing one of the first studies to show a direct link between the source and fate of salt marsh macrophyte carbon.

What we have learned from these studies is that there are often strong associations between the physical and chemical features of the environment, the composition of the microbial communities, and their activities, but the rules that govern these associations have not been fully elucidated. Krause et al. argue in this Research Topic that using a combination of ecophysiology approaches and contemporary molecular tools to develop a trait-based framework, microbial ecologists have an opportunity to forge new understandings of biodiversity and ecosystem function relationships and to generate systematic principles that apply to both micro- and macro-ecology. This Research Topic focuses on studies that address the role of microbial diversity in ecosystem processes, with emphasis on direct and quantitative links between biodiversity and processes in natural systems.

## Author contributions

All authors listed, have made substantial, direct and intellectual contribution to the work, and approved it for publication.

### Conflict of interest statement

The authors declare that the research was conducted in the absence of any commercial or financial relationships that could be construed as a potential conflict of interest.

## References

[B1] Bernhard A. E. Tucker J. Giblin A. E. Stahl D. A. (2007). Functionally distinct communities of ammonia-oxidizing bacteria along an estuarine salinity gradient. Environ. Microbiol. 9, 1439–1447. 10.1111/j.1462-2920.2007.01260.x 17504481

[B2] Cavigelli M. A. Robertson G. P. (2000). The functional significance of denitrifier community composition in a terrestrial ecosystem. Ecology 81, 1402–1414. 10.1890/0012-9658(2000)081[1402:TFSODC]2.0.CO;2

[B3] Kelly J. J. Policht K. Grancharova T. Hundal L. S. (2011). Distinct responses in ammonia-oxidizing archaea and bacteria after addition of biosolids to an agricultural soil. Appl. Environ. Microbiol. 77, 6551–6558. 10.1128/AEM.02608-10 21803892 PMC3187164

[B4] Philippot L. Spor A. Henault C. Bru D. Bizouard F. Jones C. M.. (2013). Loss in microbial diversity affects nitrogen cycling in soil. ISME J. 7, 1609–1619. 10.1038/ismej.2013.34 23466702 PMC3721106

[B5] Webster G. Embley T. M. Freitag T. E. Smith Z. Prosser J. I. (2005). Links between ammonia oxidizer species composition, functional diversity and nitrification kinetics in grassland soils. Environ. Microbiol. 7, 676–684. 10.1111/j.1462-2920.2005.00740.x 15819850

[B6] Wittebolle L. Marzorati M. Clement L. Balloi A. Daffonchio D. Heylen K.. (2009). Initial community evenness favours functionality under selective stress. Nature 458, 623–626. 10.1038/nature07840 19270679

