# Addendum: Editorial: Linking Ecosystem Function to Microbial Diversity

**DOI:** 10.3389/fmicb.2016.01299

**Published:** 2016-08-22

**Authors:** Anne E. Bernhard, John J. Kelly

**Affiliations:** ^1^Biology Department, Connecticut CollegeNew London, CT, USA; ^2^Biology Department, Loyola University ChicagoChicago, IL, USA

**Keywords:** metatranscirptomics, permafrost, fungi, decomposition, diversity

Two additional papers that are featured in this Research Topic are published in Frontiers in Terrestrial Microbiology. Valentin et al. (2014) explore relationships of fungal diversity and respiration rates of decomposing wood. And, using metatranscriptomics to investigate microbial activities in thawing permafrost, Coolen and Orsi (2015) demonstrate a potential link between bacteria carrying out acetogenesis and methanogenesis.

## Author contributions

Both authors consulted on and drafted the commentary.

### Conflict of interest statement

The authors declare that the research was conducted in the absence of any commercial or financial relationships that could be construed as a potential conflict of interest.
